# Characterization of YhcN in stress adaptation and its complex transcriptional regulation by SlyA in *Yersinia pestis*

**DOI:** 10.3389/fcimb.2026.1769634

**Published:** 2026-03-16

**Authors:** Yumeng Wei, Kai Song, Jiali Shi, Jiamin Li, Yanping Han, Zongmin Du, Ruifu Yang, JianQin Zhou, Yajun Song

**Affiliations:** 1 College of Pharmaceutical Sciences, Soochow University, Suzhou, China; 2 State Key Laboratory of Pathogen and Biosecurity, Academy of Military Medical Sciences, Beijing, China

**Keywords:** SlyA, stress adaptation, transcriptional regulation, *Yersinia pestis*, YhcN

## Abstract

SlyA, a key transcriptional regulator in *Yersinia pestis*, is involved in stress adaptation and virulence, yet its regulatory mechanisms remain poorly understood. Here, we investigate the role of the conserved hypothetical protein YhcN and its regulation by SlyA in *Y. pestis*. We demonstrate that deletion of *yhcN* impairs bacterial viability under low-temperature and anaerobic conditions and growth. Intriguingly, both deletion and overexpression of *slyA* lead to upregulation of *yhcN* transcription, suggesting a complex regulatory interplay. Bioinformatics identified two SlyA-binding sites upstream of the predicted -35 box of the *yhcN* promoter. We propose that SlyA may mediate a complex regulation of *yhcN*, wherein basal SlyA level could repress the transcription of the *yhcN*, while elevated SlyA could activate it. This regulatory switch may enable *Y. pestis* to fine-tune YhcN expression under fluctuating environmental pressures. Taken together, our findings propose a novel and complex regulatory circuit involving SlyA and YhcN, advancing the understanding of transcriptional networks that govern stress adaptation in *Y. pestis*.

## Introduction

*Yersinia pestis*, the causative agent of plague, employs a sophisticated regulatory network to control the expression of genes in response to diverse and hostile environments within its vector and hosts (Barbieri et al., 2020; Yang et al., 2023). Among the key transcriptional regulators involved in bacterial adaptation and pathogenicity is SlyA (also termed RovA in certain bacteria), a member of the MarR (multiple antibiotic resistance regulator) family (Ellison and Miller, 2006; Deochand and Grove, 2017). SlyA homologs are widely distributed in bacteria and participate in diverse cellular processes, including oxidative stress response, antimicrobial peptide resistance, and virulence gene regulation (Nagel et al., 2003; Cabezas et al., 2018; Liu et al., 2018a; Will et al., 2019; Will and Fang, 2020). SlyA/RovA has been identified as a global transcription factor in *Y. pestis*, contributing to its virulence through regulating virulence genes such as *psaEFABC* (Cathelyn et al., 2006). Notably, SlyA also acts as a negative regulator of biofilm formation, while its K73Q modification significantly promotes biofilm formation of *Y. pestis* (Liu et al., 2016; Tan et al., 2023). Furthermore, deletion of the *slyA* gene in *Y. pestis* results in attenuated bacterial virulence on mice, confirming the role of *slyA* in regulating the pathogen’s pathogenicity (Cathelyn et al., 2006; Yang et al., 2010).

Interestingly, genomic studies have revealed that *slyA* harbors mutation hotspots within *Y. pestis* populations, most of which are nonsynonymous or nonsense mutations (Mas Fiol et al., 2024; Wu et al., 2025). These variations suggest potential functional diversification or adaptation of SlyA across different ecological niches, particularly in response to environmental stresses. That is, SlyA may repress specific stress-response genes under certain conditions, an underexplored aspect of its regulatory repertoire.

A putative target for such regulation is the gene *yhcN* (YP_0565), which encodes a conserved hypothetical protein containing a DUF1471 domain, also known as YdgH/BhsA/McbA-like domain (pfam07338) (Driscoll et al., 2014; Chuanboon et al., 2019). YhcN has been implicated in bacterial stress response in several enteric pathogens. For example, in *Escherichia coli*, YhcN is involved in acid resistance and biofilm formation, though its regulatory mechanisms remain elusive (Hancock et al., 2010; Driscoll et al., 2014). In *Y. pestis*, transcriptomic studies indicated that *yhcN* family genes were upregulated in a *phoP* mutant during flea infection, and overexpression of *yhcN* was shown to enhance bacterial survival under acidic conditions (Vadyvaloo et al., 2015). Microarray analysis revealed that deletion of *rovA/slyA* leads to significant upregulation of *yhcN* transcription in both *Y. pestis* and *Yersinia enterocolitica* (Cathelyn et al., 2006, 2007). Furthermore, transcriptomic data available through the Yersiniomics database (Lê-Bury et al., 2023) suggest that the *yhcN* upregulated when the *crp*, *cobB*, or *yfiQ* was deleted, or cultured in macrophage cells or plasma (Chauvaux et al., 2007; Fukuto et al., 2010; Liu et al., 2018b; Ritzert et al., 2019), while downregulated when cultured in TMH with glucose as carbon source compared to glycerol as carbon source (Ritzert et al., 2019). Despite these suggestive links, a systematic functional characterization of YhcN and the molecular basis of its regulation in *Y. pestis* and other *Enterobacteriaceae* has been lacking.

In this study, we demonstrate the important role of YhcN in *Yersinia pestis* adaptation to key environmental stresses, including low temperature and anaerobiosis. We further uncover a non-canonical, complex regulatory relationship mediated by the global regulator SlyA: both deletion and overexpression of *slyA* result in significant upregulation of *yhcN* transcription. The regulatory pattern of SlyA on *yhcN* transcription suggests a possible complex regulatory mechanism for *yhcN* transcription regulated by SlyA. Collectively, this work establishes YhcN as a crucial stress-adaptation factor and elucidates a previously unrecognized layer of complex transcriptional control by SlyA, advancing our understanding of how bacteria dynamically rewire gene expression to survive in fluctuating environments.

## Results

### *yhcN* deletion causes a growth defect in *Yersinia pestis*

To assess the contribution of YhcN to bacterial fitness, we compared the growth of the wild-type strain 201 and the isogenic Δ*yhcN* mutant in two media: nutrient-rich LB and a chemically defined TMH medium (**Supplementary Table 1**). In both media, the Δ*yhcN* mutant exhibited a consistent growth defect relative to the wild type (**Supplementary Figures 1A, B**). And the *yhcN* complementary strain recovered the growth in LB medium. Quantitative comparison of the normalized area under the growth curve (AUGC) and the doubling times of the wild-type and the Δ*yhcN* mutant confirmed that the overall growth capacity of the mutant was significantly reduced in LB and in TMH medium (**Supplementary Figure 1C**).These results indicate that YhcN supports efficient growth of *Y. pestis* across diverse nutrient environments, suggesting its function extends beyond stress tolerance to include roles in general metabolic fitness or cellular homeostasis.

### *yhcN* deletion impairs bacterial survival under stress conditions

To investigate the role of YhcN in stress resistance, we compared the survival of the Δ*yhcN* mutant and the wild-type strain under several conditions relevant to the *Y. pestis* life cycle.

**Figure 1:**
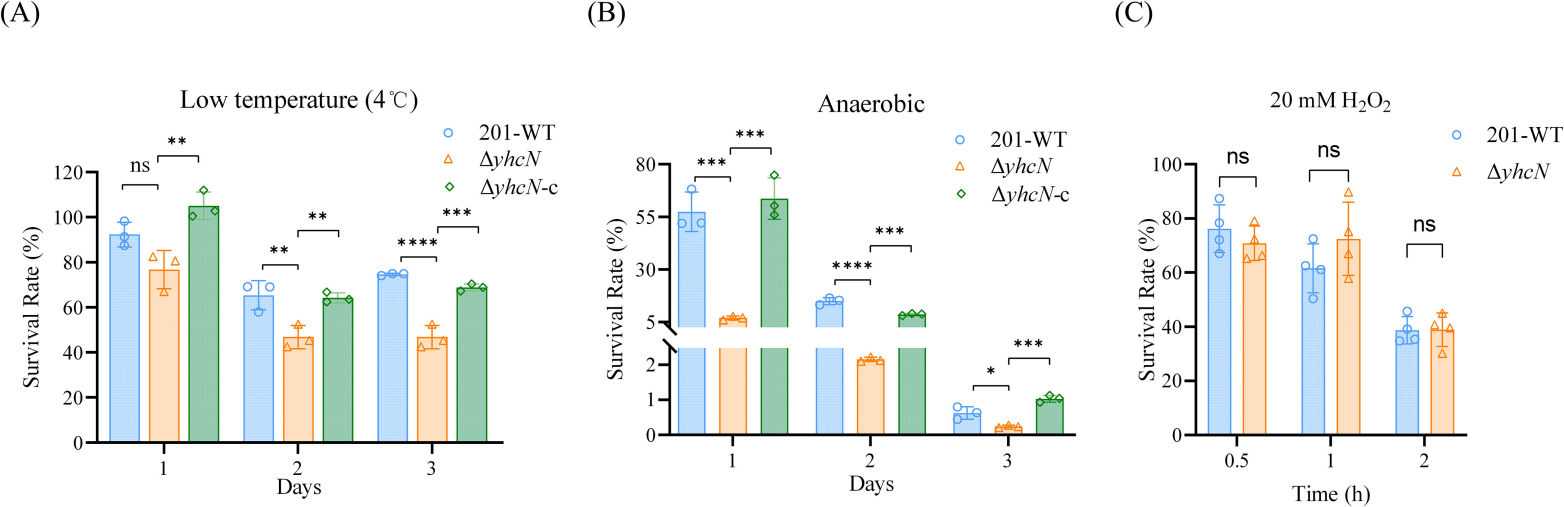
Phenotypic characterization of the Δ*yhcN* mutant under stress. Survival rates of wild-type (WT), *ΔyhcN* and *ΔyhcN*-c strains after exposure to: **(A)** 4 °C for 3 days; **(B)** anaerobic conditions for 3 days. **(C)** Survival rates of WT and *ΔyhcN* strains after exposure to 20 mM H_2_O_2_ for the indicated times. Data are mean ± SD of three independent experiments with four replicates each. **P < 0.01, ***P < 0.001, ****P < 0.0001 (one-way ANOVA or Student’s t-test). ns, not significant.

When challenged with low temperature (4°C) for 3 days, the survival rate of the Δ*yhcN* mutant was significantly lower than that of the wild type (**Figure 1A**). Similarly, under anaerobic conditions for 3 days, the mutant showed a marked survival defect (**Figure 1B**). The complemented strain exhibited a phenotype identical to that of the wild-type strain. Survival remained comparable between strains after treatment with 20 mM H_2_O_2_ for up to 2 h (**Figure 1C**).

Together, these data demonstrate that YhcN is important for resisting low temperature and anaerobiosis, but is dispensable for tolerance to hydrogen peroxide.

### *yhcN* deletion results in loss of cellular integrity

To visually assess cellular integrity under stress, we performed SEM on wild-type and Δ*yhcN* mutant cells after 24 h of exposure to low temperature (4°C) or anaerobic conditions, comparing them to control cultures grown under standard conditions (26°C, aerobic).

**Figure 2:**
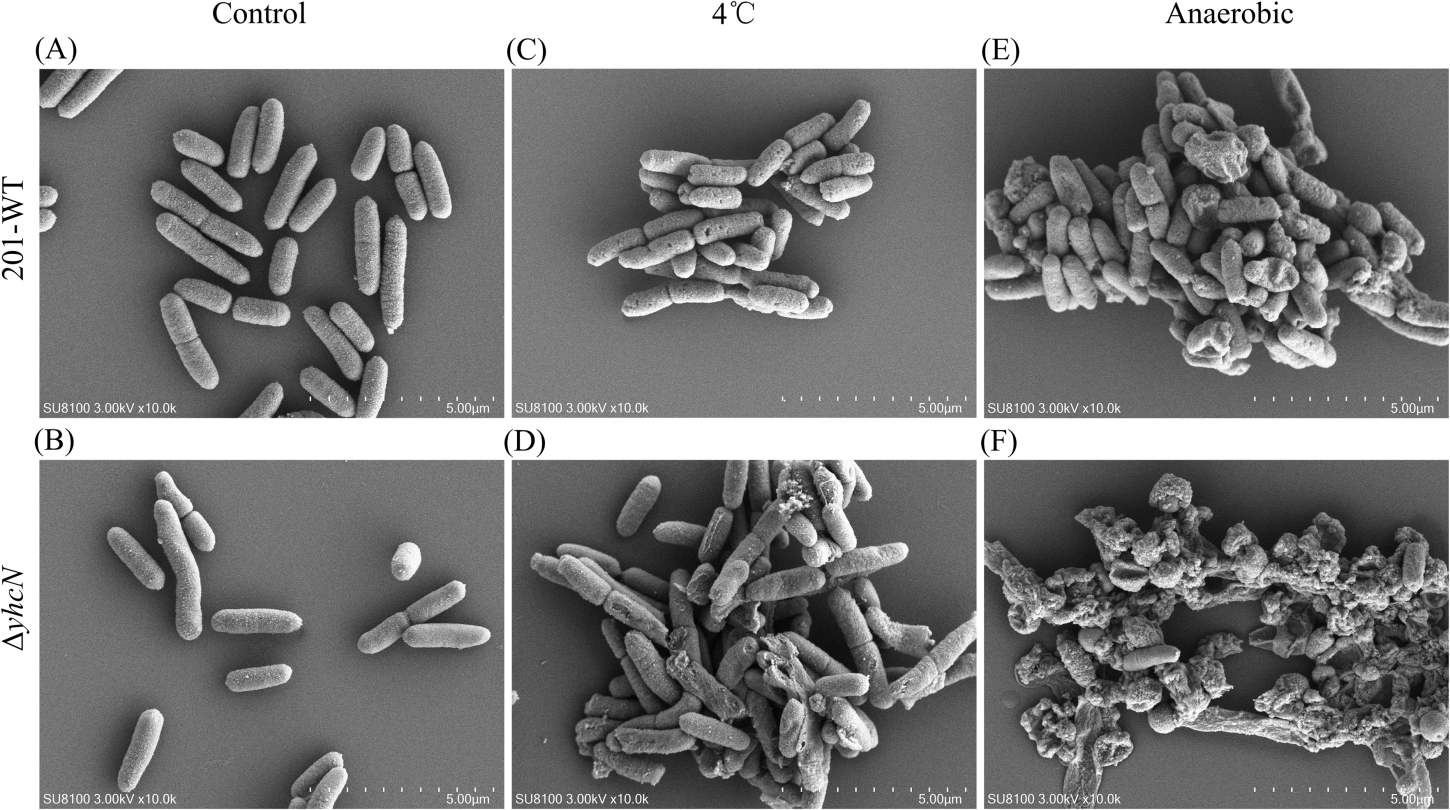
Scanning electron microscopy (SEM) of bacterial morphology under stress. Representative SEM images of WT and Δ*yhcN* cells after 24 h under the indicated conditions: **(A, B)** standard aerobic culture at 26 °C; **(C, D)** low-temperature stress (4 °C); **(E, F)** anaerobic stress. Images were captured at 10,000× magnification; scale bar = 5 µm. Three independent biological replicates were prepared for each treatment group.

Under standard conditions, both strains exhibited typical rod-shaped morphology with smooth, intact surfaces (**Figures 2A, B**). Following low-temperature stress, wild-type cells showed signs of surface roughness and minor morphological alterations, but the majority of cells remained intact and recognizable (**Figure 2C**). In contrast, the Δ*yhcN* mutant population displayed widespread and severe damage, including cell shrinkage, membrane invagination, and apparent rupture of a substantial fraction of cells (**Figure 2D**).

The morphological disparity was even more pronounced after anaerobic stress. While wild-type cells maintained a largely coherent structure despite some surface irregularities (**Figure 2E**), the Δ*yhcN* mutant exhibited extreme loss of cellular integrity, characterized by extensive cell collapse, lysis, and debris formation (**Figure 2F**).

These SEM observations provide direct visual evidence that YhcN is critical for maintaining cellular structural integrity during cold and anaerobic adaptation. The severe morphological defects in the Δ*yhcN* mutant align with its significantly reduced viability under the same conditions, strongly supporting the conclusion that YhcN plays a vital role in preserving membrane and/or cell wall stability under these specific environmental pressures.

### Low-temperature and anaerobic stress induce coordinated upregulation of *slyA* and *yhcN*

Given the impaired survival of the Δ*yhcN* mutant under low-temperature and anaerobic conditions, we asked whether these stresses also affect the expression of *slyA* and *yhcN* in the wild-type strain. Using qRT-PCR, we monitored transcript levels of both genes after shift to 4°C or to anaerobic incubation.

**Figure 3:**
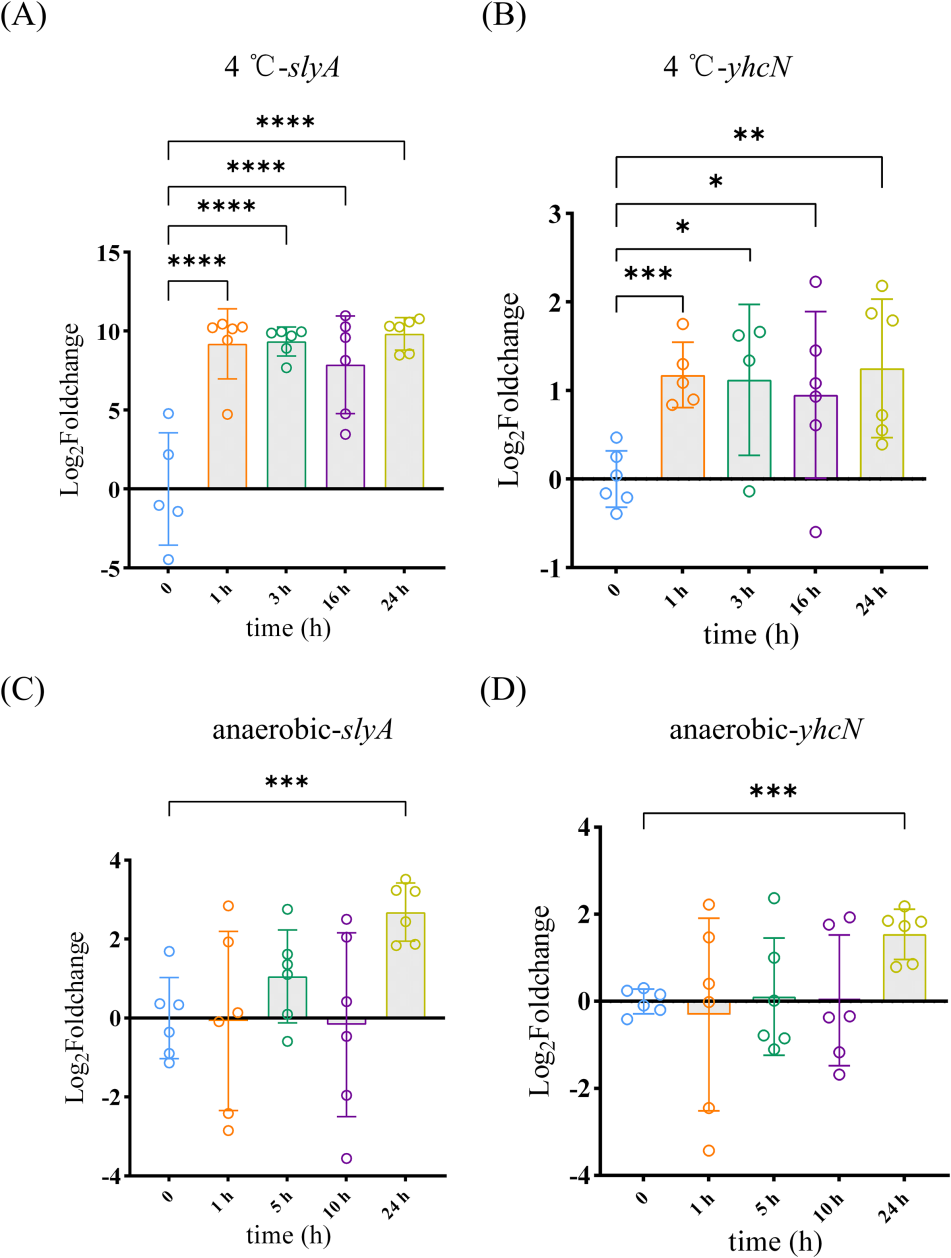
Stress-induced co-upregulation of *slyA* and *yhcN*. qRT-PCR analysis of *slyA***(A, C)** and *yhcN***(B, D)** transcript levels in WT *Y. pestis* under **(A, B)** low-temperature (4 °C) stress over a 24-h time course, and **(C, D)** after 24 h of anaerobic culture. Data are expressed as log_2_ fold-change relative to the respective unstressed control (26 °C or aerobic), normalized to *rpoB*, and represent mean ± SD of three independent experiments with six replicates total. The variability of control stems from biological replicates, and each data point was normalized to the mean of all control replicates. *P < 0.05, **P < 0.01, ***P < 0.001, ****P < 0.0001 (one-way ANOVA).

Under low-temperature stress, *slyA* expression was significantly induced at 1, 3, 16, and 24 h compared with the control maintained at 26°C (**Figure 3A**). Correspondingly, *yhcN* transcript levels rose in parallel throughout the time course (**Figure 3B**).

A similar co-induction pattern was observed under anaerobic conditions. After 24 h of anaerobic culture, both *slyA* and *yhcN* mRNA levels were markedly higher than those in the aerobic control (**Figures 3C, D**).

These results indicate that the same environmental stresses that require YhcN for full bacterial survival also trigger the concerted transcriptional upregulation of *slyA* and *yhcN*. This co-induction pattern is consistent with *yhcN* being part of an adaptive response to these conditions, potentially under the influence of SlyA.

### Transcription of *yhcN* is associated with SlyA levels

**Figure 4:**
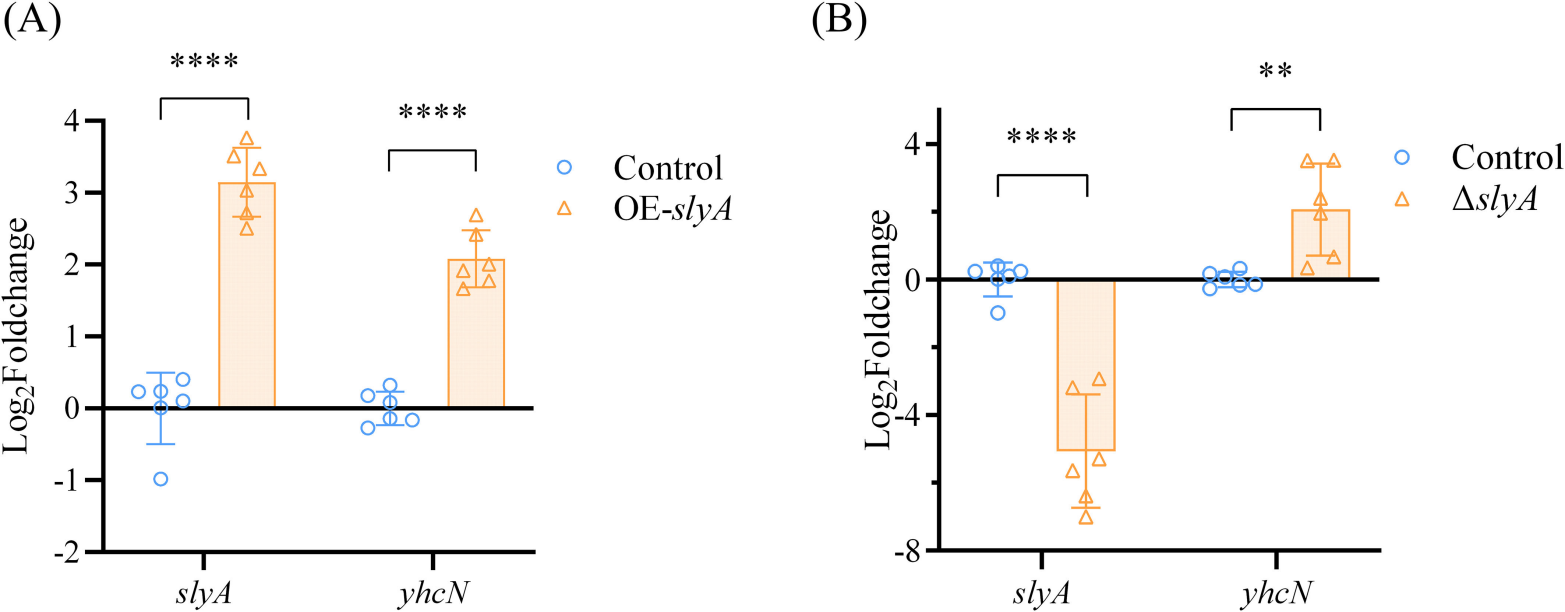
SlyA levels bidirectionally regulate *yhcN* transcription. **(A)** qRT-PCR analysis of *slyA* and *yhcN* transcript levels in the SlyA-overexpression strain (OE-*slyA*) relative to WT. **(B)** qRT-PCR analysis of *slyA* and *yhcN* transcript levels in the Δ*slyA* mutant relative to the wild-type (WT) strain. The variability of control stems from biological replicates, and each data point was normalized to the mean of all control replicates. Data are normalized to *rpoB* and presented as mean ± SD of three independent experiments with six replicates total. **P < 0.01, ****P < 0.0001 (Student’s t-test).

The coordinated upregulation of *slyA* and *yhcN* under low-temperature and anaerobic prompted us to ask whether SlyA directly regulates *yhcN* expression. To test this, we first examined *yhcN* transcript levels upon SlyA overexpression. In the OE-*slyA* strain (overexpression of the *slyA*), where *slyA* transcript was markedly induced, *yhcN* expression was robustly upregulated compared to the wild type (**Figure 4A**). This result confirms that elevated SlyA levels can activate *yhcN* transcription (directly or indirectly), aligning with the co-induction pattern observed under stress.

If SlyA were a simple activator, its absence should reduce or abolish *yhcN* expression. Contrary to this expectation, RT-qPCR analysis of the Δ*slyA* mutant revealed a significant increase in *yhcN* transcript levels relative to the wild type (**Figure 4B**). This phenotype confirms and extends an earlier microarray observation that the transcription level of yhcN is significantly upregulated in a *slyA* null mutant of *Y. pestis* (Cathelyn et al., 2006) and demonstrates that basal SlyA activity under non-stress conditions acts to repress *yhcN*.

Collectively, these data reveal a complex regulatory relationship between SlyA and *yhcN*: *yhcN* expression is heightened both when SlyA is absent and when it is in surplus. This non-linear response suggests that SlyA may function as a concentration-dependent switch at the *yhcN* promoter.

### Bioinformatics analysis of YhcN protein homology and the *yhcN* promoter

**Figure 5:**
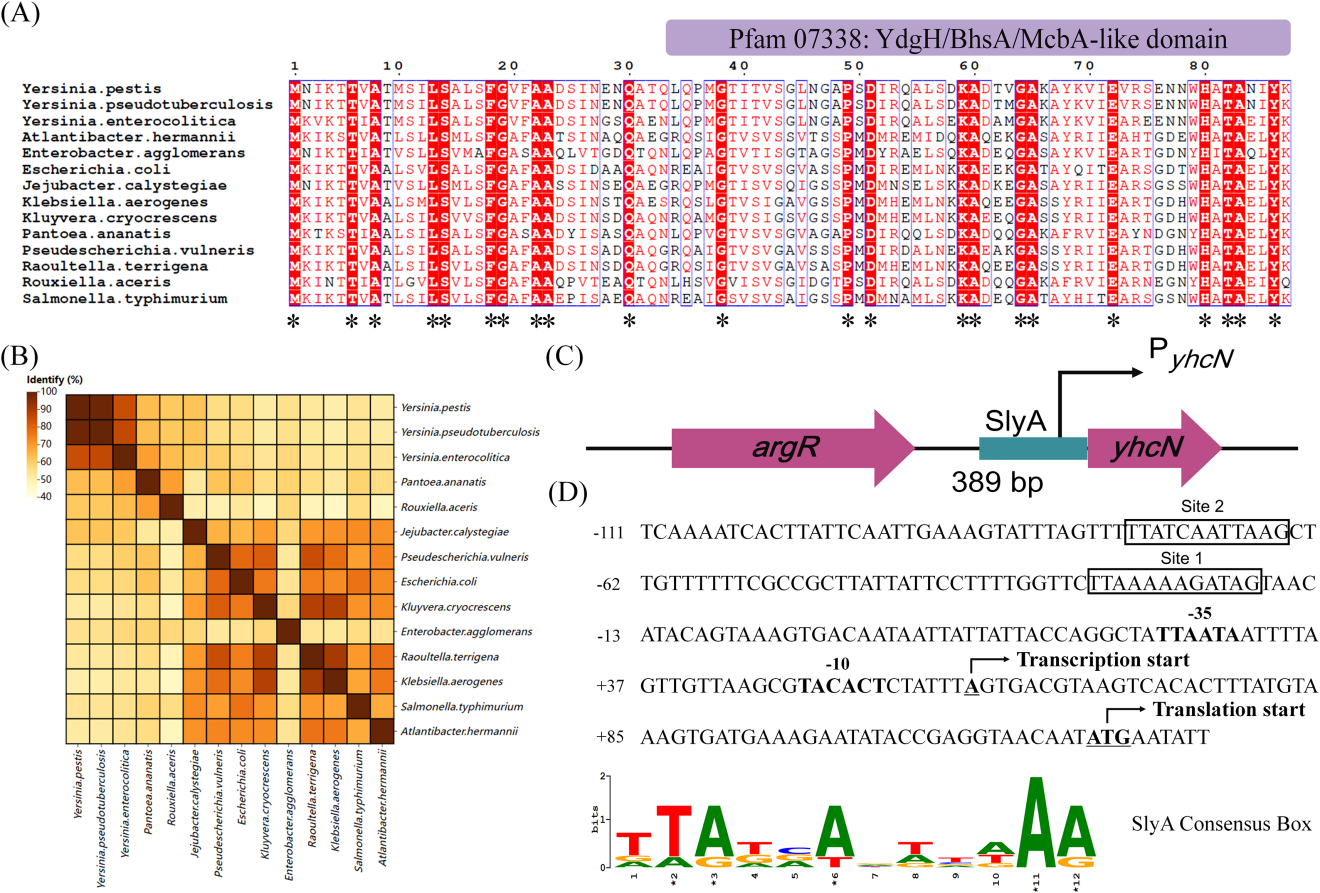
Bioinformatic analysis of YhcN and its promoter. **(A)** Multiple sequence alignment of YhcN homologs from representative *Enterobacteriaceae* strains. Identical and conserved residues are highlighted in red background and light red text, respectively. The conserved YdgH/BhsA/McbA-like domain (PF07338) is indicated. **(B)** Heatmap depicting pairwise sequence identity of YhcN across the 14 strains analyzed. **(C)** Schematic of the genomic context of *yhcN*, showing the upstream gene (*argR*) and the candidate promoter region. The black arrow indicates the predicted transcription start site (TSS). **(D)** Detailed view of the *yhcN* promoter region. Key elements include the TSS, −10 and −35 boxes, and two predicted SlyA-binding sites (Site 1 and Site 2).

We further performed a multiple sequence alignment of YhcN amino acid sequences from 14 representative strains from *Enterobacteriaceae*. The alignment revealed high conservation, including a central YdgH/BhsA/McbA-like domain (Pfam 07338) (**Figure 5A**). A within-group correlation heatmap further showed that the *Y. pestis* YhcN shares at least 51.72% identity with homologs from other enteric bacteria (**Figure 5B**). These analyses confirm that YhcN is a member of the conserved DUF1471 family, which has been implicated in bacterial stress responses (Driscoll et al., 2014). Unfortunately, there is no available YhcN PDB files, which keep us away from the structure annotation. We retrieved existing predicted structure of YhcN from UniProt predicted by AlphaFold, showing the protein consists of two distinct structural regions: a flexible N-terminal extension and a globular C-terminal domain (**Supplementary Figure 2**). The N-terminal region (colored yellow to reddish) is composed of disordered coils and a short α-helix, providing high flexibility to span spatial distances or interact with membrane surfaces. The C-terminal domain (colored blue to cyan) adopts a compact α/β fold, featuring a combination of α-helices and β-sheets that form a stable globular architecture, which typically houses the functional core of the protein, such as an enzyme active site or a protein-protein interaction interface.

To explore the potential for direct regulation by SlyA, we analyzed the *yhcN* promoter. A 389-bp region upstream of the *yhcN* coding sequence was defined as the candidate promoter (**Figure 5C**). A transcription start site at bp –56 relative to the start codon was predicted according to the transcriptomic profiling works on *Yersinia pseudotuberculosis* performed by Nuss et al (Nuss et al., 2015), along with putative –10 and –35 boxes (**Figure 5D**).

We then scanned this region for SlyA-binding motifs using the *Escherichia coli* SlyA consensus (PRODORIC accession MX000236) and the FIMO tool. Two putative SlyA-binding sites were detected: one located 42 bp upstream of the –35 box (Site 1), and the other situated 89 bp upstream of the –35 box (Site 2) (**Figure 5D**). Site 1 displayed 58.3% identity to the SlyA consensus box in *Salmonella enterica* Serovar Typhimurium (Ballesteros et al., 2019), while Site 2 showed 50% identity, suggesting that Site 1 might possess higher binding affinity.

### SlyA protein binds to the *yhcN* promoter *in vitro*

To validate the bioinformatic predictions, we assessed the direct binding of SlyA to the *yhcN* promoter *in vitro* using electrophoretic mobility shift assays (EMSAs). A 371-bp PCR fragment encompassing the predicted promoter region with both putative SlyA-binding sites (P*yhcN*) was used as the probe.

**Figure 6:**
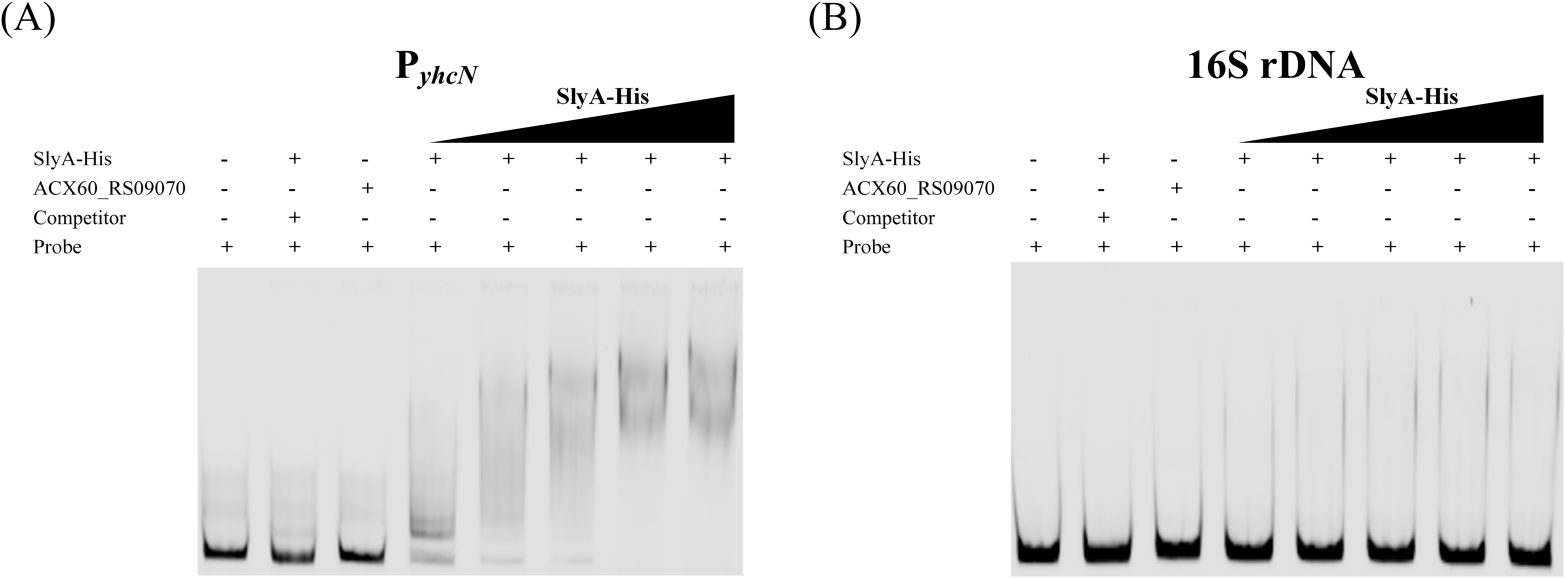
SlyA binds specifically to the *yhcN* promoter *in vitro*. Electrophoretic mobility shift assay (EMSA) showing binding of purified SlyA-His protein to the *yhcN* promoter probe (P*yhcN*). **(A)** Lanes: 1, probe only (~ 8.1 nM); 2, probe + SlyA-His + 10× unlabeled competitor probe (~ 81 nM); 3, probe + unrelated control protein (ACX60_RS09070); 4–8, probe + increasing concentrations of SlyA-His (0.070, 0.140, 0.209, 0.279 and 0.349 µg). **(B)** Control EMSA using a 16S rDNA probe under the same conditions.

As shown in **Figure 6**, incubation of the purified SlyA-His protein with the labeled P*yhcN* probe resulted in a concentration-dependent gel shift, indicating the formation of a protein-DNA complex (**Figure 6A**). In contrast, no shift was observed when SlyA was incubated with a labeled 16S rDNA fragment used as a negative control (**Figure 6B**). The specificity of the interaction was confirmed by a competition assay, where the addition of an excess of unlabeled *yhcN* competitor probe effectively reduced the intensity of the shifted band (**Figures 6A**, lane 2).

The EMSA results demonstrate that SlyA specifically binds to the *yhcN* promoter region *in vitro*. This provides biochemical evidences for the direct transcriptional regulation of *yhcN* by SlyA and corroborates the presence of at least one binding site.

## Discussion

The conserved hypothetical protein YhcN, a member of the widespread DUF1471 domain family (Pfam07338), has been associated with stress responses in enteric bacteria, including contributions to acid resistance and biofilm formation in *Escherichia coli* (Hancock et al., 2010; Driscoll et al., 2014). Transcriptional analysis revealed that, two YhcN family genes, y0666 (the ortholog of *yhcN* studied here) and y1667 were significantly upregulated in the *ΔphoP* mutant *Y. pestis* KIM6+ strain during flea infection, suggesting their roles in the adaptation within the flea vector (Vadyvaloo et al., 2015). However, a systematic functional characterization of YhcN and the molecular basis of its regulation in *Y. pestis* remained unresolved. Here, we demonstrate that YhcN is a critical fitness factor for *Y. pestis* under specific environmental stresses and delineate a complex transcriptional regulatory circuit orchestrated by the global regulator SlyA.

Our phenotypic analysis reveals a dual role for YhcN in basal growth and stress-specific adaptation. The growth defect of the Δ*yhcN* mutant in both rich and chemical defined media (**Supplementary Figure 1**) indicates a fundamental function in cellular metabolism or homeostasis, potentially underpinning its ability to mitigate stress. More definitively, YhcN is essential for survival under two key environmental pressures: low temperature, and anaerobiosis (**Figures 1A, B**). The severity of this requirement is vividly illustrated by scanning electron microscopy, which demonstrates that in the absence of YhcN, cells subjected to these stresses undergo catastrophic structural failure—marked by widespread membrane invagination, shrinkage, and lysis—phenotypes largely absent in the stressed wild type (**Figure 2**). This morphological collapse aligns with previous findings that deletion of *rovA/slyA* alters the expression of cell envelope genes and compromises the membrane integrity (Yang et al., 2010). This strongly suggests that YhcN, a critical yet selective fitness determinant, plays a vital role in preserving cell envelope integrity under these specific physical and chemical challenges.

Notably, our data failed to link YhcN with the resistance to oxidative stress (H_2_O_2_) (**Figure 1C**), which differs from the finding in *Escherichia. coli* (Lee et al., 2009), suggesting potential species-specific functional diversification. Additionally, while acid stress was included in our initial screening, we observed considerable variability in survival outcomes across independent biological replicates, precluding a definitive conclusion about YhcN’s role under this condition (not shown). Future work will be needed to elucidate the potential role of YhcN in the acid stress response of *Y. pestis*.

As well known, SlyA is a MarR family regulator involved in diverse stress and virulence responses (Will and Fang, 2020). It is known that SlyA regulates several target genes in *Y*. *pestis*, such as *hmsT*, *psaABC*, *psaEF*, and *slyA* (Liu et al., 2016; Tan et al., 2023). Accordingly, SlyA negatively regulates biofilm formation, activates pH6 antigen expression, and plays a role in pathogenicity (Cathelyn et al., 2006; Yang et al., 2010; Tan et al., 2023). Nevertheless, the precise mechanisms by which SlyA regulates individual target genes in response to environmental cues remain poorly defined. SlyA/RovA is a well-established thermoregulated virulence activator at 37°C (Quade et al., 2012). Intriguingly, we found that the same conditions which necessitate YhcN for survival, low temperature and anaerobiosis, also trigger the coordinated upregulation of both *slyA* and *yhcN* (**Figure 3**), suggesting its regulation is more complex. This co-induction suggests the SlyA–YhcN axis as an integral, inducible component of the transcriptional adaptive response to these environmental challenges. We speculate that this dual induction might serve as a pre-adaptive strategy: sensing non-thermal stresses in the flea gut (like anaerobiosis) could prematurely induce SlyA and its regulon (including *yhcN*), potentially preparing the bacterium for the impending temperature shift and hostile conditions encountered upon transmission to the mammalian host. The precise mechanism underlying this low-temperature induction and its integration with thermoregulation is indeed a compelling question for our future studies.

Contrary to a simple linear relationship, both genetic ablation and overexpression of *slyA* led to significant upregulation of *yhcN* (**Figure 4**). This paradoxical phenotype suggested a regulatory mechanism capable of producing opposite outputs—repression and activation—depending on SlyA levels. Similar biphasic or dual-function regulation is known for other global regulators like CRP, functioning not only as a transcriptional activator for carbon metabolism genes but also as a repressor of its own gene (*crp*) and specific targets like *ompA*, forming a self-feedback mechanism (Aiba, 1983; Geng and Jiang, 2015; Sonnleitner, 2025). Furthermore, SlyA could inhibit the expression of RcsB in *S. enterica* Serovar Typhimurium by directly binding to two putative binding sites in promoters of the *rcsB*, with differential binding affinities (higher for P*_rcsDB_*, lower for P*_rcsB_*) (Ballesteros et al., 2019).

We propose tentative model of concentration-dependent regulation for *yhcN*. In this model, under basal conditions, the limited SlyA molecules likely occupy the binding site of *yhcN*, which may slightly hinder the binding of the RNA polymerase via a steric hindrance and exert weak repression. Upon stress-induced (or experimental) overexpression, the increased SlyA concentration might recruit another regulatory factor to form a complex with SlyA, which could play a role on the recruit of the RNA polymerase and activate the transcription of the *yhcN*. Although consistent with our data, this model remains speculative and serves only as a working hypothesis. The binding sites and their individual roles associated to the regulatory switch await experimental identification and delineation. Future studies employing site-directed mutagenesis in reporter constructs and quantitative assays like ChIP under varying SlyA levels will be essential to rigorously define the mechanism. If validated, this elegant regulatory logic would enable *Y. pestis* to minimize the fitness cost of constitutive YhcN expression under permissive conditions while allowing its rapid induction in harsh environments like low temperature or anaerobiosis, thereby fine-tuning adaptive fitness.

In summary, our work defines YhcN as a crucial adaptation factor for *Y. pestis* against key environmental stresses and elucidates a novel regulatory circuit in which SlyA acts as a concentration-dependent transcriptional switch. The proposed switch might represent a sophisticated strategy for balancing the potential fitness cost of constitutive YhcN expression with the need for rapid induction under fluctuating pressures. While our model is consistent with genetic, transcriptional, and biochemical data, future studies employing site-directed mutagenesis of each binding site are required to definitively assign their individual roles in repression and activation. Furthermore, elucidating the precise biochemical function of the conserved DUF1471 domain in YhcN will be key to understanding how this protein preserves membrane integrity and promotes survival. Collectively, these findings significantly advance our understanding of the transcriptional networks that underpin stress adaptation and environmental persistence in this formidable pathogen.

## Materials and methods

### Bacterial strains and culture conditions

The bacterial strains and plasmids used in this study are listed in **Table 1**, with primer sequences. *Yersinia pestis* strain 201 possesses a genome identical to strain 91001; it is highly lethal in mice but avirulent in humans (Song et al., 2004; Han et al., 2005). *Y. pestis* was grown in LB (Luria-Bertani) medium at 26°C and *Escherichia coli* was cultured in LB medium at 37°C. Antibiotics were supplemented when required: ampicillin (100 µg/mL) for strains carrying the pBAD24 plasmid, and chloramphenicol (34 µg/mL) for strains harboring the pDS132 plasmid. All procedures involving live *Y. pestis* were conducted within a certified biological safety cabinet.

**Table 1:** Bacterial strains and plasmids, primers used in the study.

Bacterial strains and plasmids	Genotype	Reference
E. coli		
S17λpir	Tp^r^ Sm^r^*recA thi pro hsdR^−^M^+^* (RP4-2-Tc::Mu: Kan^r^Tn7) λpir	Philippe et al., 2004
S17-pDS132-*yhcN*-del	pDS132-*yhcN*-del was introduced into S17λpir	This study
S17-pDS132-*slyA*-del	pDS132-*slyA*-del was introduced into S17λpir	This study
DH5α	F-φ80lacZΔM15 Δ(*lacZYA*-arg F) U169 endA1 *recA1 hsdR17*(rk−, mk+) *supE*44 λ- thi-1 *gyrA96* relA1 *phoA*	Xiao et al., 2023
DH5α-pBAD24-*yhcN*	pBAD24-*yhcN* was introduced into DH5α	This study
BL21(DE3)	F-ompT *hsd*SB(rB ^−^mB ^−^) gal dcm (DE3)	Xiao et al., 2023
BL21(DE3):: pET28a-*slyA*	To express the protein SlyA-His	This study
Y. pestis		
201-WT	*Y. pestis* biovar Microtus strain 201, WT	Han et al., 2005
Δ*yhcN*	deleted *yhcN* based on strain 201-WT	This study
Δ*slyA*	deleted *slyA* based on strain 201-WT	This study
OE-*slyA*	201-WT with overexpression of *slyA*	This study
Δ*yhcN*-comp	Δ*yhcN* with pBAD24−*yhcN*	This study
Plasmids		
pDS132	Improvement of pCVD442, a suicide plasmid for gene allele exchange in bacteria	Philippe et al., 2004
pBAD24-*slyA*	To overexpress of *slyA*	This study
pBAD24-*yhcN*	To complement *yhcN*	This study
pET28a (+)	Overexpression vectors, carry an N-terminal His-Tag/T7-Tag configuration plus an optional C-terminal His-Tag sequence, Kan^r^	Xiao et al., 2023
Primer	Primer sequence (5’-3’)	Primer function
Construction of 201-ΔyhcN		
*yhcN*-F (1134)	CTTCTAGAGGTACCGCATGCTTAGGTAAAGCAGAGGGG	Amplify the homology arm of *yhcN.*
*yhcN*-R	TGGAATTCCCGGGAGAGCTCGGGTTATGCAAAAATGAG	
pDS132-F (1369)	TGAACGGCAGGTATATGTG	Identify whether the recombinant vector is constructed successfully
pDS132-R	CGTTACATCCCTGGCTTGTT	
Seq-*yhcN*-F (711)	TTAAACACCGAGCAGTAAAATG	Identify whether the *yhcN* is knocked out successfully.
Seq-*yhcN*-R	GATAAAAAAGCAGAACAAGCAA	
RT-qPCR		
q-*slyA*-F (196)	ACCACCAGAGCAATCACAGA	Amplify *slyA* internal sequence.
q-*slyA*-R	ACAAATCACGCCATCAACCT	
q-*yhcN*-F (58)	ATAAAGTGATTGAAGTGCGT	Amplify *yhcN* internal sequence.
q-*yhcN*-R	TTGTAAATATTAGCGGTGGC	
q-*rpoB*-F (188)	GCCTTGGGTGATTTTACG	Amplify *rpoB* internal sequence.
q-*rpoB*-R	CGGTTCTGGTTTCTGGTG	
EMSA		
16S-EMSA-F (230)	GACAAAGACTGACGCTCAGG	Amplify the negative probe sequences to generate both unlabeled and 5’-Alexa Fluor 680-labeled products.
16S-EMSA-R	CGTTGCATCGAATTAAACCA	
E-yhcN-F (371)	TCATAGCCTTCACCCTCA	Amplify the *yhcN* promoter region to generate both unlabeled and 5’-Alexa Fluor 680-labeled products.
E*-yhcN*-R	GCTGCAAAAACACCAAAT	

The underlined bases indicate the restriction enzyme sites.

The bracketed numbers indicate expected fragment size for the product.

### Sequence analysis of YhcN and the *yhcN* promoter region

The YhcN protein (NP_995209.1) studied here is encoded by YP_0565 of *Y. pestis* strain 91001 (genome accession number AE017042.1). For comparative analysis, YhcN amino acid sequences from 14 representative *Enterobacteriaceae* strains were retrieved from the Taxonomy database (https://www.ncbi.nlm.nih.gov/guide/taxonomy/). Multiple sequence alignment was performed using CLUSTALW (https://www.genome.jp/tools-bin/clustalw) and visualized with (https://espript.ibcp.fr/ESPript/ESPript/) (Gouet, 2003; Hall, 2013). The conserved YdgH/BhsA/McbA-like domain (PF07338) was annotated based on Pfam database (https://www.ebi.ac.uk/interpro/entry/pfam/) and Conserved Domain Databases (https://www.ncbi.nlm.nih.gov/cdd). A heatmap depicting sequence identity correlation of YhcN was generated using Chiplot online tool (https://www.chiplot.online/). The promoter region of *yhcN* was analyzed using a 389-bp sequence upstream of its coding start site. Putative promoter elements, including the transcription start site, -10 box, and -35 box, were predicted according to the mTSS prediction using transcriptomic profiling, which suggested a ~56–57 nt 5’-UTR for the *yhcN* in *Y. pestis* and *Y. enterocolitica* (Nuss et al., 2015; Schmühl et al., 2019). To identify potential SlyA-binding sites, the *yhcN* promoter sequence was scanned against the *E. coli* SlyA consensus motif (PRODORIC accession MX000236) using PRODORIC and FIMO (https://meme-suite.org/meme/tools/fimo) (Grant et al., 2011; Dudek and Jahn, 2022).

### DNA extraction, amplification and mutant construction

Genomic DNA was extracted using the QIAamp DNA Mini Kit, and plasmid DNA was purified with the QIAprep Spin Miniprep Kit, following the manufacturer’s protocols. Target DNA fragments were amplified by standard PCR.

### Construction of the mutants

The plasmid pDS132 was digested with SphI-HF and SacI-HF at 37°C for 4 hours (Philippe et al., 2004). Homology arms for *yhcN* were ligated into the linearized vector using 2×Seamless Cloning Mix at 50°C for 15 min (vector: fragment molar ratio 1:3). The recombinant plasmid was transformed into *E. coli* S17λpir to generate S17-pDS132-*yhcN*-del for subsequent conjugation with *Y. pestis*.

S17-pDS132-*yhcN*-del was grown in LB at 37°C to OD_600_ 0.4-0.8, while *Y. pestis* 201-WT was grown in LB at 26°C to OD_620_ of 0.8. Cells were harvested by centrifugation (2,600 × *g*, 5 min). Pellets from 1 mL donor and 100 µL recipient cultures were resuspended, mixed, and spotted onto a 0.22 µm filter membrane placed on an LB agar plate. After overnight incubation at 26°C, cells were washed from the filter and plated on *Yersinia* Selective Agar Base (YSAB) supplemented with chloramphenicol (6.8 µg/mL). Putative conjugants were selected on LB plates containing 7% sucrose. Colonies were verified by PCR and sequencing to confirm the *yhcN* deletion, yielding the Δ*yhcN* mutant. The Δ*slyA* mutant was constructed using the same procedure.

For Δ*yhcN* complementary strain construction, the plasmid pBAD24 was linearized with NheI-HF and EcoRI-HF. The *yhcN* fragment and linearized pBAD24 vector were assembled using 2× Seamless Cloning Mix (vector: insert molar ratio 1:3) at 50°C for 15 min. The assembly reaction was chemically transformed into *E. coli* DH5α competent cells. Recombinant pBAD24−*yhcN* plasmids were verified by Sanger sequencing. Purified pBAD24−*yhcN* was introduced into the Δ*yhcN* mutant by electroporation (2.5 kV, 25 µF, 200 Ω, 2 mm cuvette). Transformants were confirmed by PCR and Sanger sequencing and designated Δ*yhcN-c*.

For SlyA overexpression, the pBAD24-*slyA* plasmid was introduced into 201-WT by electroporation (2.5 kV, 25 µF, 200 Ω, 2 mm cuvette). Transformants were confirmed by PCR and sequencing and designated OE-*slyA*.

### Growth rate determination

To assess the impact of YhcN on growth, Strains 201-WT and Δ*yhcN* were grown to mid-exponential phase (OD_620_ ≈ 1.0) in LB medium at 26°C. Cultures were then diluted 1:100 into 60 mL of fresh LB or defined TMH medium (Straley and Bowmer, 1986) in Erlenmeyer flasks. For growth-curve assays, cultures of Δ*yhcN-c* were grown with ampicillin (final concentration 100 μg/mL) and L−arabinose (final concentration 5 mM). Flasks were incubated at 26°C with shaking at 200 rpm in a temperature-controlled incubator-shaker. Optical density at 600 nm (OD_600_) was measured at hourly intervals until cultures entered the decline phase. Growth curves were plotted, and the normalized area under the growth curve (AUGC) was calculated for quantitative comparison. Doubling times in LB and TMH were determined from the exponential phase by linear regression of ln (OD_600_) versus time and reported as mean ± SD. The doubling time was calculated from the exponential growth between 5–7 h in LB medium and between 6–8 h in TMH medium, using a time interval of 0.5 h.

### Survivability under stressful environments

Bacterial survival under various stress conditions was assessed by colony-forming unit (CFU) enumeration. Overnight cultures of 201-WT and Δ*yhcN* strains were standardized to an OD_620_ of 1.0 (approximately 2 × 10^8^ CFU/mL). For survivability assays, cultures of Δ*yhcN-c* were maintained throughout the experiment in LB supplemented with ampicillin (100 μg/mL) and L−arabinose (5 mM). Cultures were centrifuged, washed three times with PBS, adjusted to OD_620_ ≈ 1.0, and were then diluted 1:10 in the appropriate treatment buffer(unless otherwise noted) and subjected to the following treatments: (i) incubation at 4°C for up to 3 days (1 mL aliquots in 24-well plates; outer wells filled with PBS to reduce edge effects); (ii) anaerobic incubation for up to 3 days in anaerobic bags; (iii) exposure to 20 mM H_2_O_2_ (addition of 30% H_2_O_2_ to 1:10 diluted cells to reach a final concentration of 20 mM) for 0.5, 1 and 2 h at 26°C, 200 rpm. After each treatment, cultures were serially diluted in PBS and spot-plated onto Hiss agar. Plates were incubated at 26°C for 48 h before colonies were counted. Survival rates were calculated by comparing CFU counts after stress to counts from the untreated control. Each experiment was performed with three independent biological replicates, and results are presented as the mean ± standard deviation.

### SEM analysis

For electron microscopy, strains 201-WT and Δ*yhcN* were grown to OD_620_ 1.0 at 26°C, harvested, and washed with PBS. Cells were either fixed immediately or subjected to a 24-hour intervention under specified conditions prior to fixation. Then the fixed samples were processed and imaged by Servicebio Co., Ltd. (Wuhan, China). For each experimental condition (standard, low-temperature, anaerobic), samples were prepared from three independent biological cultures to ensure reproducibility.

### Real-time quantitative reverse transcription PCR RT-qPCR

Strains Δ*slyA* and OE-*slyA* were grown in LB at 26°C to OD_620_ = 1.0. Cells were pelleted (2,600 × *g*, 5 min), washed three times with PBS, and used for RNA extraction. Total RNA was extracted following resuspension of the pellet in 100 μL of lysozyme solution. Reverse transcription was performed to generate cDNA, followed by dilution of the product to 5 ng/μL. The transcriptional levels of *slyA* and *yhcN* in the Δ*slyA* and OE-*slyA* strains were measured by RT-qPCR using the primers listed in **Table 1**, with expression normalized to *rpoB*.

Gene expression in 201-WT was also analyzed under low-temperature and anaerobic conditions using the same RNA extraction and RT-qPCR protocol. RT-qPCR data were analyzed by one-way ANOVA with Tukey’s post-hoc multiple-comparisons test; P-values < 0.05 were considered significant.

### Expression and purification of SlyA

To obtain recombinant SlyA protein, the *slyA* coding sequence was inserted into the pET28a (+) vector, generating a construct for expression of an N-terminally His-tagged fusion. The plasmid was introduced into *E. coli* BL21(DE3). Protein expression was induced with 0.5 mM IPTG when cultures reached an OD_600_ of 0.6–0.8, followed by incubation at 16°C for 15 h.

Cells were harvested by centrifugation, resuspended in lysis buffer (50 mM NaH_2_PO_4_, 300 mM NaCl, 10 mM imidazole, pH 7.0) with protease inhibitor, and lysed by sonication. The soluble fraction was obtained by centrifugation at 10,000 × *g* for 5 min at 4°C.

SlyA-His was purified from the supernatant by nickel-affinity chromatography using Ni-NTA resin. After washing with buffer containing 20 mM imidazole, the protein was eluted with 250 mM imidazole. The eluted protein was desalted and concentrated. Purity and identity were verified by SDS-PAGE and Western blot, and protein concentration was determined using a spectrophotometer.

### Electrophoretic mobility shift assay

To validate the *in vitro* binding of SlyA to the *yhcN* promoter, electrophoretic mobility shift assays were performed as previously described (Xiao et al., 2023). The target promoter region (P*yhcN*, 371 bp) and a control 16S rDNA fragment were amplified by PCR. For the binding reactions, a 5′-AlexaFluor 680-labeled P*yhcN* probe (40 ng) was incubated with increasing concentrations of purified SlyA-His protein in a 20 µL reaction mixture containing 1× binding buffer, 50 mM KCl, 5 mM MgCl_2_, 5% glycerol, 0.05% NP-40, and 5 ng/µL Salmon Sperm DNA. Specificity controls included reactions with an unrelated protein (ACX60_RS09070) and competition with a 10-fold molar excess of unlabeled P*yhcN* probe. After incubation at room temperature for 20 min in the dark, reactions were loaded onto a 4% native polyacrylamide gel and electrophoresed at 90 V for 1 h at 4°C in 0.5× TBE buffer. Fluorescently shifted bands were visualized immediately using an infrared imaging system.

## Data Availability

The original contributions presented in the study are included in the article/**Supplementary Material**. Further inquiries can be directed to the corresponding authors.

## Supplementary material

Supplementary Figure 1 Growth curves of WT and Δ*yhcN* strains. **(A)** Growth of WT, Δ*yhcN*, and Δ*yhcN*-c strains in LB medium at 26°C. **(B)** Growth of WT and Δ*yhcN* mutant in the defined TMH medium at 26°C. **(C)** Doubling times of the WT and Δ*yhcN* mutant in LB and TMH medium. Insets show the normalized area under the growth curve (AUGC) for each strain. Data are mean ± SD of four biological replicates from two independent experiments. *P < 0.05, **P < 0.01 (Student’s t-test or one-way ANOVA).

Supplementary Figure 2 Predicted structure of the YhcN retrieved from Uniprot predicted by AlphaFold. **(A)** Model confidence of the predicted YhcN structure. **(B)** The domain of the predicted YhcN structure (in green).
