# Supplementary material for: Characterization of YhcN in stress adaptation and its complex transcriptional regulation by SlyA in Yersinia pestis
Source: Front Cell Infect Microbiol. 2026 Mar 16;16:1769634. doi: 10.3389/fcimb.2026.1769634 (PMC13033631; doi:10.3389/fcimb.2026.1769634)
Supplement: Supplementary file 1 [file Table1.docx]

Supplementary Table 1 Defined TMH medium

|  | Ingredients (MW) | Storage fluid (mM) | Concentration (mM) |
| --- | --- | --- | --- |
| Salt solution | K_2_HPO_4_·3H_2_O (228.22) | 25.0 | 2.5 |
|  | [Citric acid](javascript:;) (192.14) | 100.0 | 10.0 |
|  | NH_4_Cl (53.49) | 100.0 | 10.0 |
|  | MgCl_2_·6 H_2_O (203.30) | 200.0 | 20 |
|  | MnCl_2_·4 H_2_O (197.92) | 0.1 | 0.01 |
| Stable amino acid solution | DL-Alanine (89.09) | 5 | 2.5 |
|  | L-Leucine (131.2) | 2 | 1.0 |
|  | L-Tyrosine (181.19) | 2 | 1.0 |
|  | L- Isoleucine (131.2) | 2 | 1.0 |
|  | L-Valine (117.15) | 2 | 1.0 |
|  | L-Phenylalanine (165) | 2 | 1.0 |
|  | L-Threonine (119.13) | 5 | 2.5 |
|  | L-Histidine (155.16) | 2 | 1.0 |
|  | L-Arginine (174) | 2 | 1.0 |
|  | L-Aspartate (133.11) | 2 | 1.0 |
|  | L-Methionine (149.21) | 2 | 1.0 |
|  | L-Proline (115.14) | 10 | 5.0 |
|  | L-Lysine (146.19) | 2 | 1.0 |
|  | L-Glutamic acid (147.13) | 10 | 5.0 |
|  | Glycine (75.07) | 10 | 5.0 |
|  | L-Serine (105.09) | 10 | 5.0 |
|  | heated in a water bath at 100 ℃ to facilitate dissolution | | |
| Unstable amino acid solution | L-Tryptophan (204.23) | 10 | 0.1 |
|  | L-Asparagine (150.13) | 125 | 2.5 |
|  | L-Glutarnine (146.15) | 50 | 1.0 |
|  | Be heated in a water bath at 60°C to dissolution and be prepared fresh before use. | | |
| Vitamin solution | V_B1_ (337.3) | 3 | 0.003 |
|  | V_B5_ (238.3) | 4 | 0.004 |
|  | V_H_ (244.3) | 2 | 0.002 |
| Others (g/L) | Na_2_S_2_SO_3_·5 H_2_O (248.17) | 0.620425 g | 2.5 |
|  | HEPES (239.312) | 5.9575 g | 25 |
|  | [potassium gluconate](javascript:;) (234.25) | 2.342 g | 10 |
|  | CaCl_2_ (110.984) | 0.27748 g | 2.5 |
|  | FeSO_4_·7H_2_O (278.01) | 0.027802 g | 0.1 |

Adjust the pH to 7.2. The solution was sterilized by filtration through a 0.22 μm membrane and subsequently stored at 4°C.

Supplementary Table 2 Information for the bacteria species used in the bioinformatic analysis.

| Species | Strain | accession number |
| --- | --- | --- |
| *Yersinia pestis* | 91001 | AE017042.1 |
| *Yersinia pseudotuberculosis* | FDAARGOS_581 | CP033713.1 |
| *Yersinia enterocolitica* | IP05342 | CPXJ01000034.1 |
| *Raoultella terrigena* | NCTC 9997 | LR134253.1 |
| *Klebsiella aerogenes* | KCTC 2190 | NC_015663.1 |
| *Atlantibacter hermannii* | NBRC 105704 | BAFF01000001.1 |
| *Salmonella typhimurium* | LT2 | [AE006468.2](https://www.ncbi.nlm.nih.gov/nuccore/AE006468.2/) |
| *Pantoea ananatis* | LMG 20103 | CP001875.2 |
| *Rouxiella aceris* | SAP-1 | NZ_JAADJU000000000.1 |
| *Jejubacter calystegiae* | KSNA2 | CP040428.1 |
| *Pseudescherichia vulneris* | NBRC 102420 | NZ_BBMZ01000008.1 |
| *Enterobacter agglomerans* | PNG 92-11 | NZ_QGHE00000000.1 |
| *Echerichia coli* | K12 | HG738867.1 |
| *Kluyvera cryocrescens* | NCTC 12993 | CAADJD010000018.1 |
