# Supplementary figures and images for: Characterization of YhcN in stress adaptation and its complex transcriptional regulation by SlyA in Yersinia pestis
Source: Front Cell Infect Microbiol. 2026 Mar 16;16:1769634. doi: 10.3389/fcimb.2026.1769634 (PMC13033631; doi:10.3389/fcimb.2026.1769634)

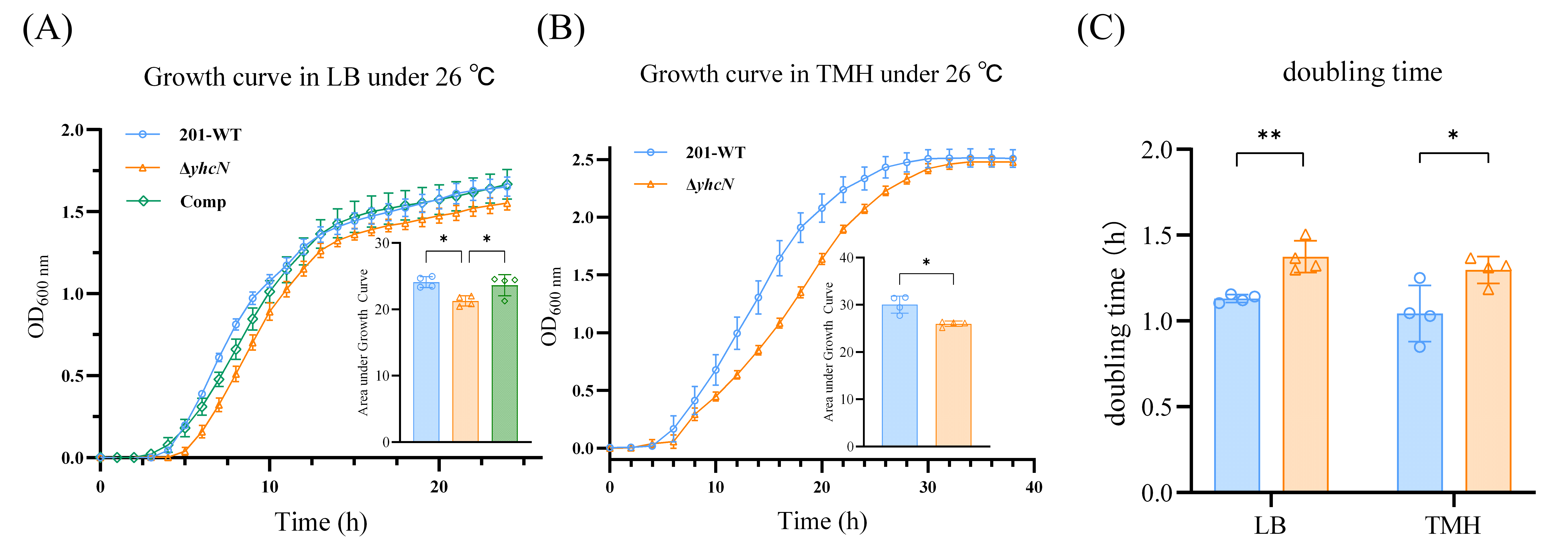

Supplement: Supplementary Figure 1 — Growth curves of WT and ΔyhcN strains. (A) Growth of WT, ΔyhcN, and ΔyhcN-c strains in LB medium at 26°C. (B) Growth of WT and ΔyhcN mutant in the defined TMH medium at 26°C. (C) Doubling times of the WT and ΔyhcN mutant in LB and TMH medium. Insets show the normalized area under the growth curve (AUGC) for each strain. Data are mean ± SD of four biological replicates from two independent experiments. *P < 0.05, **P < 0.01 (Student’s t-test or one-way ANOVA). [file Image1.tif]

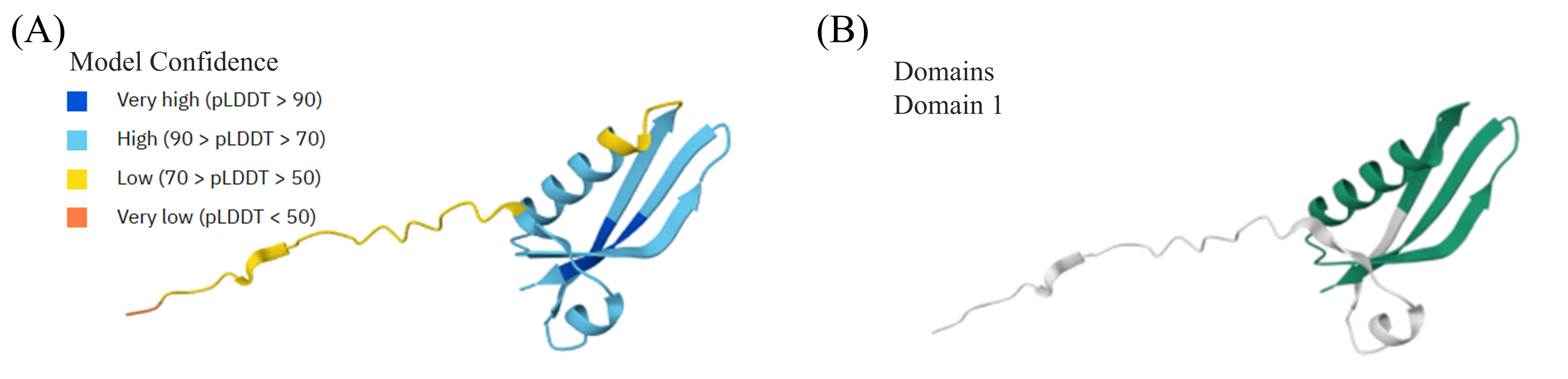

Supplement: Supplementary Figure 2 — Predicted structure of the YhcN retrieved from Uniprot predicted by AlphaFold. (A) Model confidence of the predicted YhcN structure. (B) The domain of the predicted YhcN structure (in green). [file Image2.tif]
